# DNA in honey could describe the changes in flower visits and microbe encounters of honey bees over decades

**DOI:** 10.1038/s41598-025-93315-8

**Published:** 2025-03-14

**Authors:** Alyssa R. Cirtwill, Helena Wirta

**Affiliations:** 1 Carex EcoLogics, Bracebridge, ON Canada; 2 Department of Ecology and Environmental Sciences, Umeå University, Universitetstorget 4, Umeå, Sweden; 3 Department of Agricultural Sciences, University of Helsinki, Latokartanonkaari 5, Helsinki, Finland

**Keywords:** Environmental change, Pollination, Plant, Bacteria, Fungi, *Apis mellifera*, DNA metabarcoding, Agroecology, Community ecology, Ecosystem services, Molecular ecology

## Abstract

Recent environmental changes due to land-use and climate change threaten biodiversity and the ecosystem services it provides. Understanding the true scope of these changes is complicated by the lack of historical baselines for many of the interactions underpinning ecosystem services, such as pollination, or disservices, such as disease spreading. To assess changes in such services, it is vital to find ways of comparing past and current interactions between species. Here, we focus on interactions between honey bees – one of the world’s most important agricultural pollinators, the plants they visit, and the microbes they encounter in the environment. DNA in honey offers insights into the contemporary interactions of honey bees. Old honey samples could serve to describe honey bees’ interactions in previous decades, providing a baseline against which to assess changes in interactions over time. By identifying the taxonomic origin of plant, bacterial and fungal DNA in fifty-year-old honey samples, we show that plant DNA can reveal which plants honey bees visited in the past. Likewise, microbe DNA records the microbes, including pollinator and plant pathogens, honey bees encountered and possibly spread. However, some differences in the DNA recovered between old and new honey suggest that differences in DNA degradation of different microbes could bias naive comparisons between samples. Like other types of ancient samples, old honey may be most useful for identifying interactions that historically occurred and should not be taken as proof that an interaction *did not* occur. Keeping these limits of the data in mind, time series of honey may offer unique information about how honey bees’ associations with flowers and microbes have changed during decades of environmental change.

## Introduction

Throughout human history, land-use change has impacted, and continues to impact, biodiversity and ecosystem functioning^1^. Relatively recent studies have shown climate change effects on species distributions and phenologies^2,3^, but studies investigating such effects are hampered by a general lack of baselines. To understand what has changed, we need a point of comparison from the past. In particular, while there are some long-term spatial data available from recent decades for species distributions and abundances^2^, long-term data of species interactions is close to non-existent. As interactions of species are highly important contributors to many ecosystem functions and services, including services such as pollination which are essential for human life^1^, this dearth of historical baselines is a serious problem.

Animal-mediated pollination is essential for preserving natural plant diversity as well as the quality and quantity of harvests for many important crop plants^4^. Drastic declines in pollinator populations have recently been reported, potentially threatening pollination service^5,6^. Without good historical baselines, however, we cannot really know how plant-insect interactions are changing. One of the world’s most important pollinators of cultivated plants is the honey bee, *Apis mellifera*. It is abundant worldwide^7,8^ and contributes to pollination of both wild and cultivated plants^4,9^ while it forages for nectar or pollen. Honey bees use a variety of flowering plants, but selectively visit only some plants in their local community^10–12^. Their choices impact which plants may be pollinated – as well as which other pollinators they may compete with for shared food resources^13,14^. Importantly, honey bees create a DNA record of the plants they visit in honey. Much of this DNA comes from pollen (either pollen that fell into nectar within the flower or pollen derived from a bee’s body)^15^, but there is also non-pollen plant DNA in honey^16,17^. Plant DNA in honey samples is already used to track which plants honey bees have visited and collected nectar from^10,16,18–22^. If plant DNA can also be recovered from older samples, then honey may also provide a historical record of interactions.

Together with pollen, honey bees also move microbes from one flower to another. Visits by different pollinators can shape the microbe communities in nectar^23,]^ with knock-on effects on plants, pollinators, and other animals. Bacteria, fungi, and their chemical effects have recently been shown to affect pollination rates^24,25^, as microbes (e.g. *Asaia astilbes* and *Apilactobacillus kunkeii*) living on flowers and in nectar can alter a flower’s attractiveness to pollinators^24,26–29^^23^. Apart from effects on pollination, various microbes can either protect from, or cause, disease in plants (e.g., pathogenic species in the genera *Erwinia* and *Lasiodiplodia*^30–32^) or other pollinators (including many viruses, but also bacterial and fungal pathogens, e.g. *Paenibacillus larvae* and *Melissococcus plutonius* and species of *Nosema*^24,33–36^). Even mammals can be affected by disease-causing microbes transported by honey bees, such as *Clostridium botulinum*^37^. Because of these myriad effects on plants, wild pollinators, and honey bees themselves, it is also worthwhile understanding honey bee-microbe associations and how they change over time.

The bacteria that are most strongly associated with honey bees are the ones living in their gut. There are nine bacterial species clusters found in bee guts, five of which occur in nearly all bee individuals (*Lactobacillus* Firm-4 and Firm-5, *Gilliamella apicola*, *Snodgrasella alvi* and *Bifidobacterium asteroides*) and four occurring very commonly (*Frischella perrera*, *Bartonella apis*, *Commensalibacter* sp. and *Bombella apis*)^38–41^. The gut bacteria are beneficial to honey bees and they may be shared with other insects. Honey bees also commonly host a number of bacteria that cause diseases to honey bees, such as *Paenibacillus larvae*, *Melissococcus plutonius*,* Spiroplasma melliferum* and *Serratia marcescens*^42,43^. Fungi that are commonly associated with honey bees include species which deteriorate the food stores of honey bees (e.g., yeasts in the genera *Zygosaccharomyces* and *Metschnikowia*^44,45^) and others which cause diseases in honey bees (e.g., *Ascosphora apis* and *Aspergillus niger*^46^). Recent studies have shown that DNA of these and other microbes can be recovered from honey samples^16,47–49^, allowing for comparisons of e.g. disease risk over space. As with plant DNA, if microbial DNA can be recovered from older honey samples, we may also be able to compare bee-microbe associations over time.

If plant and microbe DNA remains detectable in older honey samples, it may be possible to infer changes in honey bees’ interactions as their environment (particularly the availability of flower resources) has changed. In the case of plant DNA, differences over time may suggest changes in honey bees’ activity and choices as pollinators. While the presence of DNA in honey does not necessarily indicate pollination, a bee visiting a flower and collecting pollen is the first step towards pollination. Put simply, bees cannot pollinate plants they never visit and are more likely to pollinate plants they visit often, making the records of flower visitation preserved in honey an efficient (though admittedly imperfect) proxy for honey bee pollination. In a similar vein, though the presence of DNA of a disease-causing microbe in a honey sample does not mean that an outbreak occurred, detecting DNA of a certain microbe taxon in honey shows that honey bees have been in contact with the microbe, and may have hosted, spread, or otherwise been impacted by that microbe. If microbial DNA is highly abundant or detected in many samples, it is likely that an interaction with honey bees occurred.

So far, DNA has only been extracted from new (up to a few years old) honey samples. There is a chance that DNA may remain detectable for longer periods due to honey’s antimicrobial properties^50^, which contribute to honey being antimicrobial and thus apparently to the preservation of DNA^20,21,48^. Yet, it remains unknown how well DNA is preserved in honey over long periods. In this study, we will test this possibility in a set of four 50-year-old honey samples as a proof of concept. We aim to describe the plants from which honey bees collected nectar and which microbes they encountered, hosted and transported half a century ago by identifying plant and microbe DNA with DNA metabarcoding. To estimate the reliability of the information from DNA in old honey samples, we compare it to new honey samples.

## Materials and methods

### Samples

To describe the interactions and associations of honey bees in the past, four honey samples harvested from an apiary in South Finland five decades ago were obtained. The apiary was placed next to a large garden with both local and exotic plants, surrounded by boreal forest at 60.3°N and 24.5°E. The samples were harvested from the same apiary and by the same beekeeper in 1967, 1969, 1971, and 1972 at the end of July or the beginning of August, the end of the honey-collecting season of honey bees in Finland. The honey was packed into glass jars covered with plastic lids. The jars were stored in a shed without climate control, and the honey samples have thus experienced temperatures ranging from approximately −30 °C to +30 °C yearly for five decades. Though such temperature changes are likely to increase DNA degradation, they may also be common in older honey collected and stored before the invention of DNA barcoding and without any research objective at the time of sampling.

To compare the information from the old honey samples to information from new ones, we selected eight honey samples, each harvested by a different beekeeper in South Finland in 2019. The new samples were chosen from the same region as the old samples, less than two latitudinal and/ or longitudinal degrees from the site where the old samples originate. This region was and is a mosaic of forests and agricultural areas. All the new samples were also collected at the end of summer. They were stored in room temperature for approximately six months before being stored in the freezer until processed for DNA analyses. Both the new and the old samples are compound samples from multiple hives, each from one beekeeper.

### DNA laboratory and bioinformatic analyses

We identified taxonomic origins of the plant, bacterial, and fungal DNA in the honey samples using DNA metabarcoding, with parts of the regions ITS2 for plants and fungi and of 16S for bacteria. All the honey samples were processed together in the same way, following the DNA laboratory analyses, sequencing, and bioinformatic processing of reads as done by Tiusanen and coauthors^49^, starting from 10 g of honey. DNA was extracted with the DNeasy Plant Mini Kit (Qiagen, Germany). The targeted fragments of the specific gene regions were first amplified with tagged primers, with ITS2-F and ITS2-R^51,52^ for plants, with 16S_515FB and 16S_806RB^53,54^ for bacteria and with ITS3-KYO2 and ITS4-KYO3^55^ for fungi. The target-specific PCRs were replicated and the replicates combined for the 2nd PCR to attach unique combinatorial indices to each sample. Negative control samples were included both at the DNA extraction step as in the taxon- and gene region specific PCRs, using DNA clean water (MilliQ, Merck KGaA, Germany), and indexed the same way as other samples. All the samples were combined for sequencing, and sequenced with MiSeq sequencing runs with v3 chemistry with 600 cycles and 2 × 300 bp paired-end read length. For the bioinformatics processing the reads of all samples were combined per gene region. The reads were truncated, merged and quality controlled. Primers were removed, and the reads were dereplicated and singletons were removed. The reads were denoised to zero-radius operational taxonomic unites (ZOTUs)^56^. To remove possible misassigned reads and false positives, due to contamination, the reads in ZOTUs were filtered. As small numbers of reads were found in the controls, reads were removed if they were less than the maximum number of reads from the DNA extraction or PCR negative controls from all the samples for each ZOTU. The taxonomic assignation of ZOTUs was done by comparison against an ITS2 reference database from PLANTiTS^57^, accessed 21.3.2022, for plants, against the 16S RDP reference database^58^, version 18, for bacteria and against the UNITE fungal ITS reference database^59^, version 10.05.2021, for fungi. Taxonomic assignments were accepted with the threshold 0.9 of SINTAX probability^60^. Families and genera for which the average relative read abundance across the twelve samples considered here was less than 0.1% were omitted. Rarefaction analyses, with extrapolation, were run for plant, bacterial, and fungal ZOTUs, genera, and families. In these analyses, we used the function `iNEXT’ from the R package *iNEXT*^61,62^, to compare the accumulation curves of old and new samples.

### Statistical analyses

To test whether the number of taxa detected per sample differed between old and new honey samples, we conducted a series of two-way t-tests (one per combination of ZOTUs, genera, or families and plants, fungi, or bacteria; nine tests total). All t-tests were performed using the R v.4.3.1^63^ function “t.test” from the *stats* package^63^.

To test whether the composition of the detected communities differed between old and new honey samples, we first calculated (i) Bray-Curtis dissimilarity between vectors of presence or absence of taxa in each sample or (ii) Euclidean distance between vectors of the proportion of total reads made up by each taxon. We then tested whether old and new honey samples have similar group dispersions, as this is a prerequisite for tests of different medians. We conducted these tests using the R function “betadisper” from the package *vegan*^64^. As none of these tests indicated significantly different dispersions, we then performed PERMANOVA tests on the resulting distance matrices to test whether differences between communities were related to either sample age (new or old). We conducted these paired tests for each combination of ZOTUs, genera, or families and plants, fungi, or bacteria, as above (18 tests total, 999 permutations per test). All distance matrices were calculated using the R function “vegdist” from the package *vegan*^64^ and all PERMANOVA conducted using the function “adonis2” from the same package.

## Results

### Plant, bacterial and fungal DNA in fifty-year-old honey samples

**Fig. 1:**
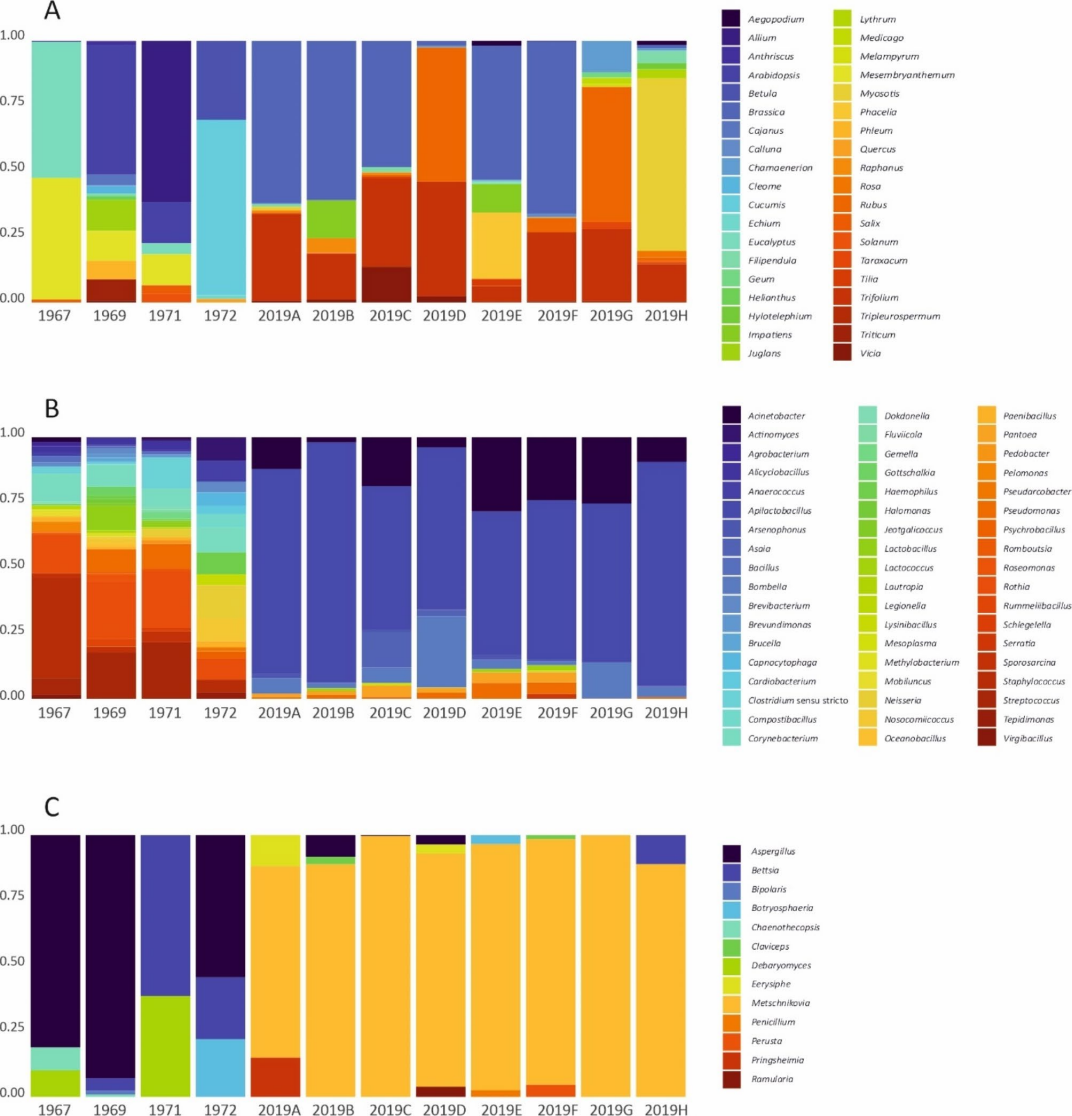
The relative read abundance of plant (**A**), bacterial (**B**) and fungal (**C**) genera identified in the honey samples examined. Each colour indicates a different genus (see legend for each panel to right).

The number of sequences obtained per honey sample after the bioinformatic processing of the reads was on average 10,336 (SD ± 516), 5318 (SD ± 2071) and 7804 (SD ± 8872) for plants, bacteria and fungi, respectively, for the fifty-year-old honey samples. Rarefaction curves reached asymptotes quickly in all cases, suggesting that increasing the sequencing depth would not impact the number of taxa detected (Fig. S1). For plants, sixty-two ZOTUs, twenty genera, and 19 families were detected in the old honey samples (Fig. 1A, Tables S1 and S2). Two of the samples had only four plant genera, while the sample collected in 1969 contained thirteen. Only two genera, *Arabidopsis* (species of which occur commonly in Finland) and *Mesembryanthemum* (species of which are used as garden plants), were found in three of the four samples. Based on the average relative read abundances, most of the plant DNA in the old honey samples originates from garden plants (genera *Mesembryanthemum*, *Cucumis*, and *Eucalyptus*), as well as *Arabidopsis* and *Allium* which could occur either as wild or planted (Table S1).

For bacteria, 249 ZOTUs, 46 genera and 36 families were detected in the old samples (Fig. 1B, Tables S3 and S4). The number of genera found per sample varied from 17 to 27, and many of the genera occurred in at least half of the samples but with low relative read abundances. Many of these are part of the gut microbiota of honey bees (e.g. *Acinetobacter*^65^, *Lactobacillus*^41^ and *Bacillus*^66^), commonly found on bee bread (*Corynoneurum*^67^) or are well-known (*Paenibacillus*^42^) or possible bee pathogens (*Haemophilus* and *Anaerococcus*^68,69^). Among genera with high frequency of occurrence there are also many that have no clear association to honey bees but are likely from the environment, such as a species of *Clostridium* which is an environmental bacterium but often found in honey^37^. Only three genera had somewhat higher average relative read abundance: *Rothia* with 15.9% (SD ± 5.3%), *Streptococcus* with 10.9% (SD ± 8.2%) and *Staphylococcus* with 10.2% (SD ± 15.0%). These three genera have also previously been detected in honey^70^. Species of *Rothia*, *Streptococcus* and *Staphylococcus* are commonly considered as opportunistic animal pathogens^71–73^, although species of *Rothia* are also found in bee guts and on bee bread^71,74^ and *Streptococcus* are also members of normal flora in many environments^72^. *Pseudomonas* and *Neisseria*, with average relative read abundances of 4.4% (SD ± 4.2%) and 4.1% (SD ± 5.1%), respectively, have also been found in honey before^70^, with species of both genera being commensals or possibly protective to animals and plants^31,75^. On top of these, many bacterial genera with no clear association to honey bees were found in lower frequencies and lower relative read abundances (Table S3).

For fungi, 44 ZOTUs, six genera, and seven families were identified from the old samples (Fig. 1C, Tables S5 and S6). Diversity of genera varied from two to four per sample. In three out of the four samples *Aspergillus* (species of which are bee pathogens^76^) and *Bettsia* (molds occurring in bee hives and living on bee bread^46^) were found, and these two genera were also the most relatively abundant based on DNA reads. Two of the four samples contained *Debaryomyces* (fungi known from bee guts and bee hives^77^) and *Chaenothecopsis* (species of which are lichens occurring in Finland^78^, though not known to be bee-associated), although with low relative read abundance.

### Comparison of plants, bacteria, and fungi detected in old and new honey samples

The new honey samples were collected as part of another study and selected here as a point of comparison after successful DNA amplification and sequencing. The number of sequences obtained from the new honey samples were on average similar to the numbers from the old samples, except for more bacterial reads being obtained from the new samples. On average 12,746 (SD ± 2148), 13,862 (SD ± 1428) and 6378 (SD ± 3257) sequences were obtained for plants, bacteria and fungi.

In total, the new samples contained 159 plant ZOTUs, 20 genera and 14 families. Significantly more plant ZOTUs per sample were detected in new honey samples than in old honey samples, but numbers of plant genera and families were not significantly different between groups (Table 1, S1, S2, S7). Plant community structure was not different for any taxonomic level, whether we considered only presence-absence data or relative read abundances. The most frequently occurring as well as the most relatively-abundant plant genera detected in the new honey samples were *Brassica*, *Trifolium*, and *Rubus*, all known to be largely used by honey bees today in Finland^79,80^, despite these genera not being detected in any of the old honey samples (Fig. 1A, Table S1). Overall, 13.2% of all the plant genera were detected in both new and old samples, and 37.5% of the plant families.

**Table 1:** Summary table of the statistical comparisons of diversity and composition of taxa among old and new honey samples, for the three taxonomic groups and the three taxonomic levels considered. ID composition refers to the presence or absence of taxa while quantitative (quant.) composition includes relative read abundance information. For detailed results, see (Table S7).

Group	Level	Richness differs	Composition (ID) differs	Composition (quant.) differs
‍Plants	ZOTU	Yes (new greater)	No	No
‍Plants	Genera	No	No	No
‍Plants	Families	No	No	No
‍Bacteria	ZOTU	No	Yes	Yes
‍Bacteria	Genera	Yes (old greater)	yes	Yes
‍Bacteria	Families	Yes (old greater)	yes	Yes
‍Fungi	ZOTU	No	yes	No
‍Fungi	Genera	No	yes	No
‍Fungi	Families	No	yes	No

The total diversity of bacteria detected in the new samples was lower than in old ones, with 122 ZOTUs, ten genera, and twelve families identified (Fig. 1B, Tables S3 and S4). The number of bacterial ZOTUs per sample did not statistically differ between old and new honey samples, but significantly more bacterial genera and families were detected in old honey samples (Table 1, S7). The composition of the bacterial community differed between old and new honey samples at all taxonomic levels, whether we considered presence-absence data or relative read abundances. The main difference in community composition is the dominance of the genus *Apiloctobacillus* in the new honey samples. On average, 68.5% (SD ± 13.4%) of the bacterial reads originated from this genus, one species of which (*A. kunkeei*) is a lactic acid bacterium living in floral nectar and in the nectar sacs of honey bees^81^ and found often to occupy most of the bacterial DNA reads in new honey samples^47,48,82^ but it was not detected in any of the old honey samples. All of the bacterial taxa identified in the new honey samples are commonly detected in honey nowadays^48^ and the genera detected included bee gut microbes, bee pathogens, and microbes protective to plants^38,42,83^.

For fungi, 101 ZOTUs, ten genera and ten families were detected in total (Fig. 1C, Tables S5 and S6). The number of fungal taxa per sample did not differ between old and new honey samples for ZOTUs, genera, or families. The composition of honey samples did, however, differ between old and new samples when considering presence-absence data, but not when including relative read abundances. This suggests that taxa with low read abundances may be driving the differences in community composition (Table 1 andS7). As for bacteria, most fungal DNA reads in the new honey samples originated from one genus. On average 90.7% (SD ± 7.8%) of the reads were from the genus *Metschnikowia*, where members of the genus are yeasts commonly occurring in bee hives, honey, and nectar^77,84^. Like *Apilactobacillus*, *Metschnikowia* was not detected in any of the old honey samples.

## Discussion

The fifty-year old honey preserved DNA of plants, bacteria and fungi. Although DNA from all three groups has previously been found in contemporary honey samples, this study is the first, to our knowledge, which demonstrates the identification of DNA in decades-old honey. Thus, honey can be added to the list of potential sources of historical DNA – complementary to the historical and ancient DNA recovered from sediments, teeth, etc^15,85–88^. With DNA that is thousands or even millions of years old being identified in the latter sources, and with identifiable DNA being preserved for decades despite the suboptimal storage conditions in our antique honey samples, we suggest that even older honey, where it is available, is worth investigating for DNA records of plant and microbial communities.

Alongside this potential, our pilot study also suggests some caveats in interpreting DNA preserved in honey with respect to changes in communities and interactions over time. Beginning with the plant DNA, we found that while the new honey samples contained significantly more plant ZOTUs than the old honey, neither the number of plant genera or families nor the composition of the communities were significantly different between age classes. In the context of Southern Finland, which has experienced environmental changes but nevertheless remains a mix of agriculture, home gardens, and boreal forest as in the 1960’s, we consider that the overall similarity of plant diversity recovered in new and old samples is encouraging. Significantly higher diversity of plant genera or families in the new samples would likely indicate that plant DNA was substantially less identifiable in the old samples, but we did not observe this. That said, two of the plant genera with highest relative abundance identified in the new samples, but not at all in the old ones, *Rubus* and *Brassica*, likely do reflect a change in honey bee foraging due to change in the environment. Finnish forest managers started to use clear-cuts, providing habitats to raspberry (*Rubus idaeus*), extensively in the 1970’s^89^. Raspberry is known to be preferred by honey bees due to its high-sugar nectar^90^, thus this difference in honey samples is likely to document a true change in honey bee foraging with increased availability of *Rubus*. Also, rapeseed (*Brassica rapa*) cultivation increased strongly in the 1970’s^91^, and thus the change shown in new and old honey samples is likely to show a real, large increase in foraging nectar from rapeseed as well. For *Trifolium*, the third plant genus with a high relative abundance of DNA and occurrence in all new honey samples but none of the old ones, we do not know of large changes in availability to honey bees in Southern Finland as different species of *Trifolium* have been cultivated in Finland for over a century^92^. Yet, it is possible that *Trifolium* was not available in the surroundings of the old apiary. Overall, as we here have old honey samples from one beekeeper and one apiary only, interpreting any differences between the old and new honey samples as showing true changes in honey bee foraging would be highly speculative. An alternative possibility is that DNA preserved in differently-structured pollen grains or as non-pollen DNA in other types of tissues and cells may degrade at different rates.

The plant DNA that was identified in the old samples, however, is from taxa that are plausibly used by honey bees. These plants are known to occur in Finland either as wild or ornamental plants^78^ and most of them are known to be currently used by honey bees in Finland^79^, so it is likely that these plants would also have been used where available in the past. Unfortunately, there is no record of which exotic plants were accessible to the honey bees which collected the nectar for the old honey, so we cannot say for certain whether each plant genus recorded is accurate. Nevertheless, honey (particularly in larger sample sizes) could provide an additional source of information on which plants were available and selected by honey bees in the past.

The greater numbers of plant ZOTUs per sample in the new honey could reflect a true increase in variation in the number of unique plant strains used by honey bees over time (noting that ZOTUs are a much finer classification than species or even variety). New varieties of garden and agricultural plants are developed every year and, with greater access to different seed sources, it is plausible that more varieties of plants used by bees are available within a given area than in past decades. Further, it is also possible that DNA degradation has a more noticeable effect at the ZOTU level than on genera or families, or that this is a statistical artifact of small sample size. Taken together, our findings for plant DNA suggest that honey bees have maintained broadly similar foraging patterns over time, despite the possible increase in usage of two currently abundantly-available plants. Yet, while plant DNA identified in old honey reflects interactions that likely did occur, the absence of a plant taxon from a sample should not be interpreted as the absence of that interaction. Note that the number of observations required to conclude a possible interaction does not occur is very large^93^ and this issue of asymmetrical information on the presence and absence of interactions (or species) is pervasive in community ecology, and thus the DNA in antique honey provides valuable information even if this information is incomplete.

Like pollen, bacterial and fungal spores are highly resistant structures to unfavourable environmental conditions. However, pollen and microbial spores vary greatly in their shapes, structures, and stress resistance^15,85,86,94^. For bacterial spores, different conditions prompt different spore structures and the durability of these spores, and the DNA preserved within, varies substantially both between and within bacterial species^85,86,95^. In the context of honey, there is no knowledge of how long DNA can be stored but it is reasonable to suppose that the variability in DNA durability mentioned above also holds true in honey. Moreover, storage times will almost certainly differ between DNA stored in pollen or spores and DNA that enters honey within small tissue pieces or single cells of the plants the honey bees visit, or as active bacterial and fungal cells from the bees themselves, the hive, flowers, and all the other parts of the environment the honey bees visit.

These caveats have clear bearing on the interpretation of the different bacterial and fungal taxa we detected in old and new honey. The genera which were most abundant in the new honey, members of the bacterial genus *Apilactobacillus* and fungal genus *Metschnikowia*, would most likely enter honey as active cells as they live in nectar, nectar sacs of honey bees^81^ or moist honey^44,45,84,96^. DNA in these active cells is likely to degrade much faster than DNA protected within spores, which could explain why these highly-abundant taxa in the new honey were not detected in the old samples.

The significant changes in bacterial diversity per sample and in bacterial and fungal composition between new and old samples could partly reflect true environmental changes but also is likely to be affected by the absence of these highly-abundant taxa after DNA in active cells has degraded. For bacteria in particular, where more bacterial genera and families were identified in the old honey samples, the absence of *Apilactobacillus* may have allowed the detection of more rare taxa. The diversity of fungi found in honey is generally smaller than that of bacteria^36,43,82^, so this same phenomenon could cause the difference in the composition of fungi observed although the diversity was not statistically different between old and new samples. This suggests that taxa with low read abundances may be driving the differences in community composition. Overall, given the generally high abundances of *Apilactobacillus* and *Metschnikowia* in new honey samples, it will be important for researchers using old honey in the future to be aware of the potential for faster DNA degradation in these and other taxa which typically enter honey as active cells rather than spores.

Other noteworthy findings include the presence of bee pathogens *Aspergillus* (e.g., *A. niger* which causes chalkbrood^76^) and *Paenibacillus* (e.g., *P. larvae* which causes American foulbrood^97^) in the focal apiary fifty years ago. These pathogens are now widespread in Finland^82^, though foulbrood was not detected in the new honey samples in this study. A wider comparison of old honey samples could be useful in understanding when and how they were initially distributed, and how more recent control measures may be affecting disease distribution. Given the small number of samples in this study and the large differences in relative read abundances of a few taxa in the new honey, we cannot definitively interpret changes in the microbes honey bees have encountered over the past decades. However, a larger sample size would provide more robust information on the microbes honey bees were, and are, exposed to. It may also be helpful to remove DNA reads of *Apilactobacillus* and *Metschnikowia* and potentially other taxa which would likely have entered honey in active form before any statistical tests in order to obtain a more apples-to-apples comparison across different honey samples (as done in a study comparing new honey samples in which the DNA of many samples mainly constituted of one very highly abundant microbe species^82^).

Ultimately, the main result of this study is the demonstration that identifiable plant, bacterial, and fungal DNA can be preserved within honey for over fifty years, even with storage temperatures ranging yearly from −30 °C to + 30 °C. The taxa we identified in the old honey samples are generally plausible nectar sources for honey bees (for plants) and include many microbes known from contemporary honey samples. We also identify an important consideration for future researchers as DNA from microbial taxa which may be active in honey dominated the reads in new samples but were absent from the old samples; further studies should take this into account by, for example, increasing read depth and ignoring reads from these taxa when statistically comparing communities. Although there have been environmental changes in South Finland during the time span of our honey samples, largely due to changes in forest management and agriculture leading to changes in the quality and quantity of flowering plants available to honey bees^98^, our sample size is simply too small to draw major ecological conclusions at this time. Nevertheless, we hope that this study will inspire researchers to examine longer time series of honey samples or larger numbers of samples, ideally stored in more favorable conditions. More expansive data could describe the change in flower visitations and microbe exposures over time and provide a detailed picture of the impacts of forestry and agricultural practices on the role of honey bees.

## Electronic supplementary material

Below is the link to the electronic supplementary material.

Supplementary Material 1

Supplementary Material 2

## Data Availability

The sequence datasets generated during the current study are available in the Sequence Read Archive repository, in the BioProject PRJNA1137582 (https://www.ncbi.nlm.nih.gov/sra/ PRJNA1137582).

## References

[1] IPBES. Summary for policymakers of the global assessment report on biodiversity and ecosystem services of the intergovernmental science-policy platform on biodiversity and ecosystem services. in *Global Assessment Report on Biodiversity and Ecosystem Services of the Intergovernmental Science-Policy Platform on Biodiversity and Ecosystem Services* (ed. Diaz, S. et al.) (IPBES secretariat, 2019).

[2] Antão, L. H. et al. Climate change reshuffles Northern species within their niches. *Nat. Clim. Chang.* **12**, 587–592 (2022).

[3] Roslin, T. et al. Phenological shifts of abiotic events, producers and consumers across a continent. *Nat. Clim. Chang.* **11**, 241–248 (2021).

[4] Klein, A. M. et al. Importance of pollinators in changing landscapes for world crops. *Proc. Royal Soc. B Biol. Sci.* **274**, 303–313 (2007). 10.1098/rspb.2006.3721 PMC1702377 17164193

[5] Murphy, J. T., Breeze, T. D., Willcox, Bryony, Kavanagh, S. & Stout, J. C. Globalisation and pollinators: Pollinator declines are an economic threat to global food systems. **4**, 773–785 (2022).

[6] Potts, S. G. et al. Global pollinator declines: trends, impacts and drivers. *Trends Ecol. Evol.* **25**, 345–353 (2010). 20188434 10.1016/j.tree.2010.01.007

[7] Global Biodiversity Information Facility. GBIF backbone taxonomy Apis mellifera. 10.15468/39omei (2024).

[8] FAO Food and Agriculture Organisation of the United Nations. FAOSTAT. https://www.fao.org/faostat/en/#home (2021).

[9] Brittain, C., Williams, N., Kremen, C. & Klein, A. M. Synergistic effects of non-*Apis* bees and honey bees for pollination services. *Proc. R. Soc. B: Biol. Sci.* 280, (2013). 10.1098/rspb.2012.2767 PMC3574329 23303545

[10] Leponiemi, M. et al. Honeybees’ foraging choices for nectar and pollen revealed by DNA metabarcoding. *Sci. Rep.* **13**, 14753 (2023). 37679501 10.1038/s41598-023-42102-4 PMC10484984

[11] De Vere, N. et al. Using DNA metabarcoding to investigate honey bee foraging reveals limited flower use despite high floral availability. *Sci. Rep.* **7**, 1–10 (2017). 28205632 10.1038/srep42838 PMC5311969

[12] Hawkins, J. et al. Using DNA metabarcoding to identify the floral composition of honey: A new tool for investigating honey bee foraging preferences. *PLoS One* **10**, (2015). 10.1371/journal.pone.0134735 PMC4550469 26308362

[13] Wojcik, V. A., Morandin, L. A., Davies Adams, L. & Rourke, K. E. Floral resource competition between honey bees and wild bees: is there clear evidence and can we guide management and conservation? *Environ. Entomol.* **47**, 822–833 (2018). 29873687 10.1093/ee/nvy077

[14] Geldmann, J. & González-Varo, J. P. Conserving honey bees does not help wildlife: high densities of managed honey bees can harm populations of wild pollinators. *Sci. (1979)*. **359**, 392–393 (2018). 10.1126/science.aar2269 29371456

[15] Sawyer, R. *Honey Identification* (Cardiff Academic, 1988).

[16] Wirta, H., Abrego, N., Miller, K., Roslin, T. & Vesterinen, E. DNA traces the origin of honey by identifying plants, bacteria and fungi. *Sci. Rep.* **11**, 4798 (2021). 33637887 10.1038/s41598-021-84174-0 PMC7910293

[17] Chavan, D. et al. Isolation and barcoding of trace pollen-free DNA for authentication of honey. *Cite This: J. Agric. Food Chem.* 14084–14095 (2022). 10.1021/acs.jafc.2c04309 36279293

[18] Valentini, A., Miquel, C. & Taberlet, P. DNA barcoding for honey biodiversity. *Divers. (Basel)* **2**, 610–617 (2010).

[19] Bruni, I. et al. A DNA barcoding approach to identify plant species in multiflower honey. *Food Chem.* **170**, 308–315 (2015). 25306350 10.1016/j.foodchem.2014.08.060

[20] Prosser, S. W. J. & Hebert, P. D. N. Rapid identification of the botanical and entomological sources of honey using DNA metabarcoding. *Food Chem.* **214**, 183–191 (2017). 27507464 10.1016/j.foodchem.2016.07.077

[21] Soares, S., Amaral, J. S., Oliveira, M. B. P. P. & Mafra, I. Improving DNA isolation from honey for the botanical origin identification. *Food Control* **48**, 130–136 (2015).

[22] Jones, L. et al. Temporal patterns of honeybee foraging in a diverse floral landscape revealed using pollen DNA metabarcoding of honey. *Integr. Comp. Biol.* **62**, 199–210 (2022). 35536572 10.1093/icb/icac029 PMC9405717

[23] Morris, M. M., Frixione, N. J., Burkert, A. C., Dinsdale, E. A. & Vannette, R. L. Microbial abundance, composition, and function in nectar are shaped by flower visitor identity. *FEMS Microbiol. Ecol.* **96**, (2020). 10.1093/femsec/fiaa003 31922546

[24] Vannette, R. L. The floral microbiome: plant, pollinator, and microbial perspectives. *Annu. Rev. Ecol. Evol. Syst.* **51**, 363–386 (2020).

[25] Steffan, S. A. et al. Microbes, the ‘silent third partners’ of bee–angiosperm mutualisms. *Trends Ecol. Evol.* **39**, 65–77 (2024). 37940503 10.1016/j.tree.2023.09.001

[26] Vannette, R. L. & Fukami, T. Contrasting effects of yeasts and bacteria on floral nectar traits. *Ann. Bot.* **121**, 1343–1349 (2018). 29562323 10.1093/aob/mcy032 PMC6007235

[27] Vannette, R., Gauthier, L., P, M. & Fukami, T. Nectar bacteria, but not yeast, weaken a plant—Pollinator mutualism. *Proc. R. Soc. B. Biol. Sci.* **280**, 20122601 (2013). 10.1098/rspb.2012.2601 PMC3574316 23222453

[28] Good, A. P., Gauthier, M. P. L., Vannette, R. L. & Fukami, T. Honey bees avoid nectar colonized by three bacterial species, but not by a yeast species, isolated from the bee gut. *PLoS One* **9**, e86494 (2014). 24466119 10.1371/journal.pone.0086494 PMC3899272

[29] Rering, C. C., Beck, J. J., Hall, G. W., McCartney, M. M. & Vannette, R. L. Nectar-inhabiting microorganisms influence nectar volatile composition and attractiveness to a generalist pollinator. *New Phytol.* **220**, 750–759 (2018). 28960308 10.1111/nph.14809

[30] Cellini, A. et al. Pathogen-induced changes in floral scent May increase honeybee-mediated dispersal of Erwinia amylovora. *ISME J.* **13:4 13**, 847–859 (2018). 10.1038/s41396-018-0319-2 PMC6461938 30504898

[31] Johnson, K. B., Stockwell, V. O., Burgett, D. M., Sugar, D. & Loper, J. E. Dispersal of *Erwinia amylovora* and *Pseudomonas fluorescens* by honey bees from hives to apple and pear blossoms. *Phytopathology* **83**, 478–484 (1993).

[32] Salvatore, M. M., Andolfi, A. & Nicoletti, R. The thin line between pathogenicity and endophytism: the case of lasiodiplodia theobromae. *Agriculture* **10**, 488 (2020).

[33] Adler, L. S., Irwin, R. E., McArt, S. H. & Vannette, R. L. Floral traits affecting the transmission of beneficial and pathogenic pollinator-associated microbes. *Curr. Opin. Insect Sci.* **44**, 1–7 (2021). 32866657 10.1016/j.cois.2020.08.006 PMC7914268

[34] Piché-Mongeon, V. & Guzman-Novoa, E. Pathogen spillover from honey bees (*Apis mellifera* L.) to wild bees in North America. *Discover Anim.* **1**, 33 (2024).

[35] Cilia, G. et al. Occurrence of honey bee (*Apis mellifera* L.) pathogens in wild pollinators in Northern Italy. *Front. Cell. Infect. Microbiol.* **12**, 907489 (2022). 35846743 10.3389/fcimb.2022.907489 PMC9280159

[36] Nanetti, A., Bortolotti, L. & Cilia, G. Pathogens spillover from honey bees to other arthropods. *Pathogens* **10**, (2021). 10.3390/pathogens10081044 PMC8400633 34451508

[37] Nevas, M. et al. High prevalence of *Clostridium botulinum* types A and B in honey samples detected by polymerase chain reaction. *Int. J. Food Microbiol.* **72**, 45–52 (2002). 11843412 10.1016/s0168-1605(01)00615-8

[38] Raymann, K. & Moran, N. A. The role of the gut Microbiome in health and disease of adult honey bee workers. *Curr. Opin. Insect Sci.* **26**, 97–104 (2018). 29764668 10.1016/j.cois.2018.02.012 PMC6010230

[39] Kwong, W. K. & Moran, N. A. Gut microbial communities of social bees. *Nat. Rev. Microbiol.* **14**, 374–384 (2016). 27140688 10.1038/nrmicro.2016.43 PMC5648345

[40] Engel, P., Martinson, V. G. & Moran, N. A. Functional diversity within the simple gut microbiota of the honey bee. *Proc. Natl. Acad. Sci. USA* **109**, 11002–11007 (2012). 22711827 10.1073/pnas.1202970109 PMC3390884

[41] Kešnerová, L. et al. Gut microbiota structure differs between honeybees in winter and summer. *ISME J.* **14**, 801–814 (2020). 31836840 10.1038/s41396-019-0568-8 PMC7031341

[42] Fünfhaus, A., Ebeling, J. & Genersch, E. Bacterial pathogens of bees. *Curr. Opin. Insect Sci.* **26**, 89–96 (2018). 29764667 10.1016/j.cois.2018.02.008

[43] Raymann, K., Coon, K. L., Shaffer, Z., Salisbury, S. & Moran, N. A. Pathogenicity of *Serratia marcescens* strains in honey bees. *mBio* 9, (2018). 10.1128/mBio.01649-18 PMC6178626 30301854

[44] Colda, A. et al. Inoculation of Pear flowers with *Metschnikowia reukaufii* and *Acinetobacter nectaris* enhances attraction of honeybees and hoverflies, but does not increase fruit and seed set. *PLoS One* **16**, e0250203 (2021). 33886638 10.1371/journal.pone.0250203 PMC8061982

[45] Liu, G. et al. Identification of *Zygosaccharomyces mellis* strains in stored honey and their stress tolerance. *Food Sci. Biotechnol.* **25**, 1645–1650 (2016). 10.1007/s10068-016-0253-x PMC6049234 30263457

[46] Evison, S. E. & Jensen, A. B. The biology and prevalence of fungal diseases in managed and wild bees. *Curr. Opin. Insect Sci.* **26**, 105–113 (2018). 29764649 10.1016/j.cois.2018.02.010

[47] Bovo, S. et al. Shotgun metagenomics of honey DNA: evaluation of a methodological approach to describe a multi-kingdom honey bee derived environmental DNA signature. *PLoS One* **13**, e0205575 (2018). 30379893 10.1371/journal.pone.0205575 PMC6209200

[48] Wirta, H., Bahram, M., Miller, K., Roslin, T. & Vesterinen, E. Reconstructing the ecosystem context of a species: Honey-borne DNA reveals the roles of the honeybee. *PLoS One* **17**, (2022). 10.1371/journal.pone.0268250 PMC9278776 35830374

[49] Tiusanen, M., Becker-Scarpitta, A. & Wirta, H. Distinct communities and differing dispersal routes in bacteria and fungi of honey bees, honey, and flowers. *Microb. Ecol.* **87**, 1–10 (2024). 10.1007/s00248-024-02413-z PMC11289361 39080099

[50] Nolan, V. C., Harrison, J. & Cox, J. A. G. Dissecting the antimicrobial composition of honey. *Antibiotics* **8**, 251 (2019). 31817375 10.3390/antibiotics8040251 PMC6963415

[51] Chen, S. et al. Validation of the ITS2 region as a novel DNA barcode for identifying medicinal plant species. *PLoS One* **5**, 1–8 (2010). 10.1371/journal.pone.0008613 PMC2799520 20062805

[52] White, T., Bruns, T., Lee, J. & Taylor, M. Amplification and direct sequencing of fungal ribosomal RNA genes for phylogenetics. in *PCR protocols: a guide to methods and applications* 315–322 (Academic Press, 1990).

[53] Walters, W. et al. Transcribed spacer marker gene primers for microbial community surveys. *mSystems* **1**, e0009–15 (2015). 10.1128/mSystems.00009-15 PMC5069754 27822518

[54] Caporaso, J. G. et al. Global patterns of 16S rRNA diversity at a depth of millions of sequences per sample. *Proc. Natl. Acad. Sci. USA* **108**, 4516–4522 (2011). 20534432 10.1073/pnas.1000080107 PMC3063599

[55] Toju, H., Tanabe, A. S., Yamamoto, S. & Sato, H. High-coverage ITS primers for the DNA-based identification of *Ascomycetes* and *Basidiomycetes* in environmental samples. *PLoS One* **7**, 40863 (2012). 10.1371/journal.pone.0040863 PMC3395698 22808280

[56] Edgar, R. UNOISE2: improved error-correction for illumina 16S and ITS amplicon sequencing. *BioRxiv* **081257**10.1101/081257 (2016).

[57] Banchi, E. et al. PLANiTS: A curated sequence reference dataset for plant ITS DNA metabarcoding. *Database* baz155 (2020). 10.1093/database/baz155 PMC6997939 32016319

[58] Cole, J. R. et al. The ribosomal database project: improved alignments and new tools for rRNA analysis. *Nucleic Acids Res.* **37**, 141–145 (2008). 10.1093/nar/gkn879 PMC2686447 19004872

[59] Nilsson, R. H. et al. The UNITE database for molecular identification of fungi: handling dark taxa and parallel taxonomic classifications. *Nucleic Acids Res.* **47**, 259–264 (2018). 10.1093/nar/gky1022 PMC6324048 30371820

[60] Edgar, R. C. SINTAX: a simple non-Bayesian taxonomy classifier for 16S and ITS sequences. 10.1101/074161

[61] Hsieh, T., Ma, K., Chao, A. & iNEXT interpolation and extrapolation for species diversity. R package version 3.0.1. (2024).

[62] Chao, A. et al. Rarefaction and extrapolation with hill numbers: A framework for sampling and Estimation in species diversity studies. *Ecol. Monogr.* **84**, 45–67 (2014).

[63] R Core Team. R: A language and environment for statistical computing. (2023).

[64] Oksanen, J. et al. Package ‘vegan’ Title Community Ecology Package Version 2.5-6. (2019).

[65] Kim, P. S. et al. Acinetobacter apis sp. nov., isolated from the intestinal tract of a honey bee, *Apis mellifera*. *J. Microb.* **52**, 639–645 (2014). 10.1007/s12275-014-4078-0 25098562

[66] Wang, M., Zhao, W. Z., Xu, H., Wang, Z. W. & He, S. Y. *Bacillus* in the guts of honey bees (*Apis mellifera*; hymenoptera: Apidae) mediate changes in amylase values. *Eur. J. Entomol.* **112**, 619–624 (2015).

[67] Piccini, C., Antúnez, K. & Zunino, P. An approach to the characterization of the honey bee hive bacterial flora. *J. Apic. Res.* **43**, 101–104 (2004).

[68] Gorrochategui-Ortega, J. et al. A short exposure to a semi-natural habitat alleviates the honey bee hive microbial imbalance caused by agricultural stress. *Sci. Rep.* **12**, 18832 (2022). 36336704 10.1038/s41598-022-23287-6 PMC9637708

[69] Hubert, J. et al. Bacteria detected in the honeybee parasitic mite *Varroa destructor* collected from beehive winter debris. *J. Appl. Microbiol.* **119**, 640–654 (2015). 26176631 10.1111/jam.12899

[70] Snowdon, J. A. & Cliver, D. O. Microorganisms in honey. *Int. J. Food Microbiol.* **31**, 1–26 (1996). 8880294 10.1016/0168-1605(96)00970-1

[71] Fatahi-Bafghi, M. Characterization of the *Rothia* spp. And their role in human clinical infections. *Infect. Genet. Evol.* **93**, 104877 (2021). 33905886 10.1016/j.meegid.2021.104877

[72] Patterson, M. J. Streptococcus. *Medical Microbiology*. (eds Baron, S.) (University of Texas Medical Branch at Galveston, 1996). 21413252

[73] Foster, T. Staphylococcus. * Medical Microbiol.*. (eds Baron, S.) (University of Texas Medical Branch at Galveston, 1996). 21413338

[74] Disayathanoowat, T. et al. Different dynamics of bacterial and fungal communities in hive-stored bee bread and their possible roles: A case study from two commercial honey bees in China. *Microorganisms* **8**, 264 (2020). 32075309 10.3390/microorganisms8020264 PMC7074699

[75] Elias, J., Frosch, M. & Vogel, U. in *Neisseria. In Manual of Clinical Microbiology*. 635–651 (eds Jorgensen, J.) 10.1128/9781555817381.ch34 (ASM, 2015).

[76] Foley, K., Fazio, G., Jensen, A. B. & Hughes, W. O. H. The distribution of *Aspergillus* spp. Opportunistic parasites in hives and their pathogenicity to honey bees. *Vet. Microbiol.* **169**, 203–210 (2014). 24485932 10.1016/j.vetmic.2013.11.029

[77] Agarbati, A., Gattucci, S., Canonico, L., Ciani, M. & Comitini, F. Yeast communities related to honeybees: occurrence and distribution in flowers, gut mycobiota, and bee products. *Appl. Microbiol. Biotechnol.* **108**, (2024). 10.1007/s00253-023-12942-1 PMC10817854 38276993

[78] Lajitietokeskus S. laji.fi. https://laji.fi/ (2022).

[79] Salonen, A., Ollikka, T., Grönlund, E. & Ruottinen, L. Julkunen-Tiitto, R. Pollen analyses of honey from Finland. *Grana* **48**, 281–289 (2009).

[80] Käpylä, M. & Niemelä, P. Flowers visited by honey bee in Southern Finland. *Agric. Food Sci.* **51**, 17–24 (1979).

[81] Olofsson, T. C. & Vásquez, A. Detection and identification of a novel lactic acid bacterial flora within the honey stomach of the honeybee *Apis mellifera*. *Curr. Microbiol.* **57**, 356–363 (2008). 18663527 10.1007/s00284-008-9202-0

[82] Wirta, H. et al. The role of seasonality in shaping the interactions of honeybees with other taxa. *Ecol. Evol.* **13**, (2023). 10.1002/ece3.10580 PMC10560870 37818248

[83] Loncaric, I. et al. Typing of *Pantoea agglomerans* isolated from colonies of honey bees (*Apis mellifera*) and culturability of selected strains from honey. *Apidologie* **40**, 40–54 (2009).

[84] Garcia-Gonzalez, M., Minguet-Lobato, M., Plou, F. J. & Fernandez-Lobato, M. Molecular characterization and heterologous expression of two α-glucosidases from *Metschnikowia* spp, both producers of honey sugars. *Microb. Cell. Fact.* **19**, (2020). 10.1186/s12934-020-01397-y PMC7353701 32652991

[85] Willerslev, E. et al. Long-Term Persistence of Bacterial DNA. 10.1016/j.cub.2003.12.012 14711425

[86] Setlow, P. & Christie, G. New thoughts on an old topic: secrets of bacterial spore resistance slowly being revealed. *Microbiol. Mol. Biol. Rev.* **87**, (2023). 10.1128/mmbr.00080-22 PMC10304885 36927044

[87] Bell, K. L. et al. Pollen DNA barcoding: Current applications and future prospects. *Genome* **59** (2016). 10.1139/gen-2015-0200 27322652

[88] von Hippel, B. et al. Long-term fungus–plant covariation from multi-site sedimentary ancient DNA metabarcoding. *Quat Sci. Rev.* **295**, (2022).

[89] Kulju, I. et al. *Metsätilastollinen Vuosikirja Finnish Statistical Yearbook of Forestry 2022* (Luonnonvarakeskus, 2023).

[90] Crane, E. & Honey (Heinemann: London, 1979).

[91] Mäkelä, P. S. A., Tuulos, A., Turakainen, M., Santanen, A. & Stoddard, F. L. Revitalizing the winter turnip rape crop in the Northern latitudes. *Acta Agric. Scand. B Soil. Plant. Sci.* **61**, 195–201 (2011).

[92] Stoddard, F. L., Hovinen, S., Kontturi, M., Lindström, K. & Nykänen, A. Legumes in finnish agriculture: History, present status and future prospects. **18** (2009).

[93] Cirtwill, A. R., Eklöf, A., Roslin, T. & Wootton, K. Gravel, D. A quantitative framework for investigating the reliability of empirical network construction. *Methods Ecol. Evol.* **10**, 902–911 (2019).

[94] Dijksterhuis, J. Fungal spores: highly variable and stress-resistant vehicles for distribution and spoilage. *Food Microbiol.* **81**, 2–11 (2019). 30910084 10.1016/j.fm.2018.11.006

[95] Setlow, P. I will survive: DNA protection in bacterial spores. *Trends Microbiol.* **15**, 172–180 (2007). 17336071 10.1016/j.tim.2007.02.004

[96] Kurtzman, C. P. DNA relatedness among species of the genus *Zygosaccharomyces*. *YEAST* **6**, 19 (1990). 10.1002/yea.320060306 2349835

[97] Genersch, E. et al. American foulbrood in honeybees and its causative agent, *Paenibacillus larvae*. *J. Invertebr Pathol.* **103**, I (2010). 10.1016/j.jip.2009.06.015 19909971

[98] Heliölä, J., Kuussaari, M. & Pöyry, J. *Pölyttäjien Tila Suomessa. Kansallista Pölyttäjästrategiaa Tukeva Taustaselvitys*.

