# Supplementary material for: DNA in honey could describe the changes in flower visits and microbe encounters of honey bees over decades
Source: Sci Rep. 2025 Mar 14;15:8807. doi: 10.1038/s41598-025-93315-8 (PMC11909190; doi:10.1038/s41598-025-93315-8)
Supplement: Supplementary file 2 — Supplementary Material 2 [file 41598_2025_93315_MOESM2_ESM.docx]

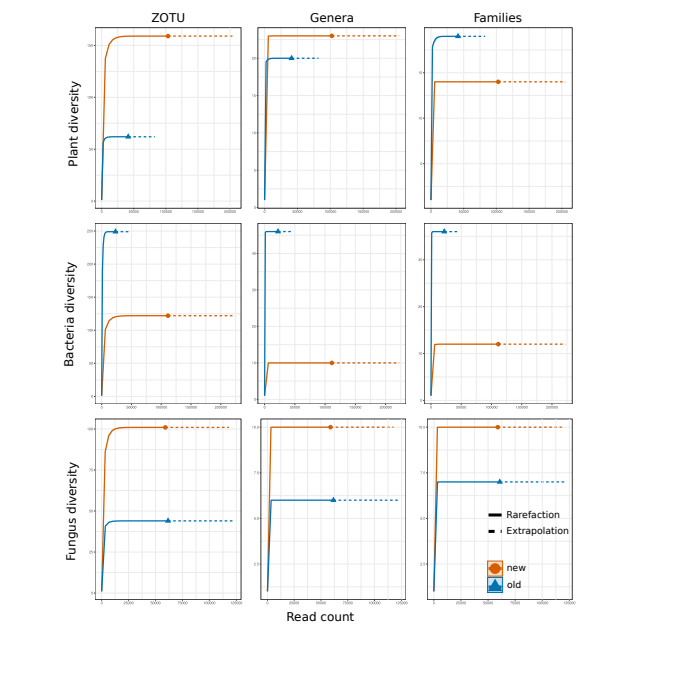
Figure S1. Rarefaction curves for each taxonomic group and level of resolution. Both old and new honey samples reached asymptotes, suggesting that increasing read depth would not increase the diversity detected.
